# Impacts of atmospheric vertical structures on transboundary aerosol transport from China to South Korea

**DOI:** 10.1038/s41598-019-49691-z

**Published:** 2019-09-10

**Authors:** Hyo-Jung Lee, Hyun-Young Jo, Sang-Woo Kim, Moon-Soo Park, Cheol-Hee Kim

**Affiliations:** 1 Department of Atmospheric Sciences, Pusan National University, Busan, 46241 Republic of Korea; 2 School of Earth and Environmental Sciences, Seoul National University, Seoul, 08826 Republic of Korea; 3 Research Center for Atmospheric Environment, Hankuk University of Foreign Studies, Yongin, 17035 Republic of Korea

**Keywords:** Natural hazards, Environmental impact

## Abstract

To forecast haze pollution episodes caused by high concentrations of long-range transported pollutants emitted in the areas upstream of South Korea, it is crucial to study and identify their behaviour. We analysed the three-dimensional air quality structure in Seoul using ground observation data and aerosol lidar measurements to identify vertical aerosol intrusion into the Korean Peninsula during the spring of 2016. The intrusions were particularly affected by the development of the atmospheric boundary layer (ABL) in the leeward regions. The nocturnal pollutant intrusion into the Korean peninsula via the Yellow Sea was examined using measured data. The pollutants first reached the area above the nocturnal boundary layer (548 ± 180 m) and approached ground level on the following day due to convective mixing depending on the convective ABL growth (1182 ± 540 m) in daytime. These intrusion mechanisms were mostly attributed to extremely high concentrations (i.e. >100 μg m^−3^) of fine particulate matter in the leeward regions, accounting for four of the total of six cases for which the warnings and alerts were issued in Seoul Metropolitan Area over a year-long period (2016). The horizontal and vertical pathways of the long-range transported pollutants and the atmospheric vertical structure were identified as key factors affecting the surface air quality concentration in the leeward regions.

## Introduction

Long-range transported dust and air pollutants emitted from China are known to affect air quality over leeward areas due to prevailing westerlies over East Asia. The impact of long-range transport of dust and air pollution poses an urgent and unresolved problem with regard to forecasting air particulate matter concentrations in Korea^1,2^ and Japan^3^ as well as understanding regional and global climate change^4–6^. Many studies have shown that natural and anthropogenic aerosols generated in China could be easily transported by westerlies to receptor areas. These results were derived from analysis of horizontal distributions of aerosol concentrations simulated by the chemical transport model as well as satellite-observed Aerosol Optical Depth (AOD) data^7–12^. Moreover, major factors affecting severe haze events in Korea have been analysed via source appointment studies^13–17^ and inverse modelling^18^. The results showed that Korea’s air quality is affected not only by local emissions but also by transboundary transport of anthropogenic emissions from China. Recent reports show that horizontal pathways of long-range transported aerosols can be determined by synoptic weather patterns^2,19^. However, the vertical structure and vertical mixing status of aerosols during long-range transport phenomena are poorly understood due to the lack of vertical measurements even though it is recognized that the altitude of the vertically mixed aerosols above the source areas and the vertical layer in which the long-range transported aerosols exist are important factors in the determination of the surface concentrations of air pollutants in leeward areas. As discussed below, in addition, understanding of the inflow mechanisms of the atmospheric vertical structure over the receptor regions is crucial if the long-range transported plumes impact the surface air quality or pass through in the upper atmosphere^20–23^. Therefore, we thoroughly examined the influence of atmospheric vertical structures related to development of the atmospheric boundary layer (ABL) on surface air quality in leeward regions. In this study, we present the observation results of a number of observed cases to investigate the vertical structures of intrusion of air pollutants generated in China into the Korean Peninsula. We employed intensive observation data, including *in-situ* observations at mountain and surface sites, and aerosol lidar measurements as we focused on meteorological conditions related to vertical mixing status over the ocean and into inland areas. The impact of the intrusion of long-range transported pollution was contrasted against the non-transported air pollutant by direct comparison between high-altitude mountains and surface sites. We also discussed the role of the development of both the marine boundary layer (MBL) and ABL in the intrusion of long-range transported air pollution and enhancement of surface pollutant concentrations.

## Results

### High PM_10_ concentrations at Mt. Gwan-ak and Seoul sites

**Figure 1:**
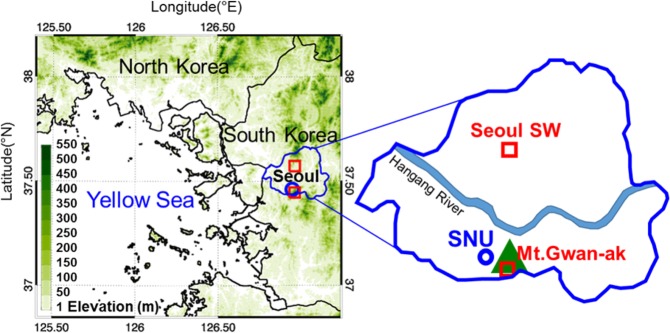
Geographical locations of PM_10_ concentrations and lidar measurements. The red squares indicate two PM_10_ concentration measurement locations: urban centre (Seoul SW) and mountain site (Mt. Gwan-ak), whereas the blue circle denotes the aerosol lidar measurement location (SNU).

**Figure 2:**
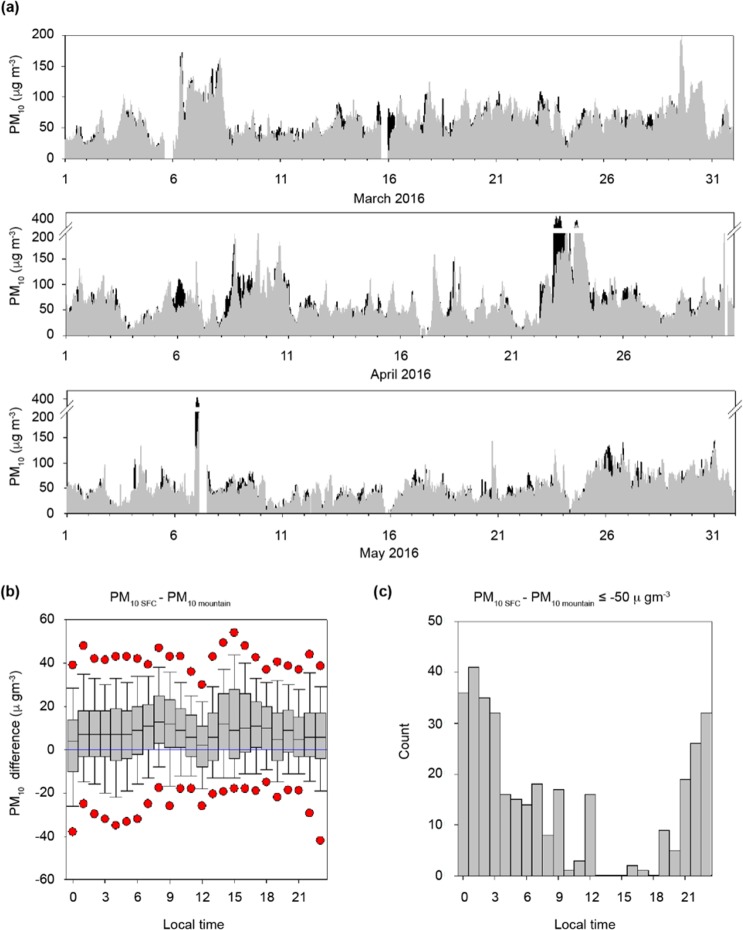
(**a**) Variations in PM_10_ concentrations measured near the surface (SFC) in Seoul (Songwol-dong) (grey bars) and at Mt. Gwan-ak (black bars) between March and May of 2016. (**b**) Distribution of PM_10_ concentration difference over time between the ground surface (Songwol-dong) and Mt. Gwan-ak in Seoul between March and May of 2016; the figure shows a percentile graph wherein the red dots represent 5/95 percent values, the error bars represent 10/90 percent values, and the grey boxes denote the 25–75 percentile ranges, wherein the black horizontal lines indicate 50 percentile values. (**c**) Number of cases in which the PM_10_ concentration difference over time between Seoul and Mt. Gwan-ak exceeded 50 μg m^−3^ between March and May of 2016.

South Korea lies on the leeward side of China and is thus heavily affected by the latter’s long-range transported pollutants, especially in spring and winter when westerly and north-westerly winds predominate. Seoul, the capital of South Korea, is particularly exposed to a variety of interlaced air pollution sources, namely local emission sources from its dense population as well as transboundary air pollution sources. Several measurements are hence being recorded to examine the behaviour of such pollutants in Seoul. The present study compares the concentration levels of particulate matter less than 10 μm in diameter (PM_10_) measured at different altitudes to visualize the pollutant behaviour in Seoul while accounting for the effects of quantified long-range transported pollutants and local emissions. *In situ* measurements of PM_10_, at various locations such as Mt. Gwan-ak at 629 m above sea level, and Song-wol at near the surface in the urban city centre were conducted, owing to the lack of previous *in situ* measurement records regarding detailed vertical profiles of PM_10_ concentrations over the Korean Peninsula and the Yellow Sea (Fig. 1). According to the time series comparison of PM_10_ concentration levels measured at Mt. Gwan-ak in the spring of 2016 against other sites in the city centre including Song-wol, PM_10_ concentration levels at Mt. Gwan-ak were often higher than those at other metropolitan surface sites (Fig. 2(a)). Although the measurement location of Mt. Gwan-ak was in the vicinity of the city’s centre, Mt. Gwan-ak, the mountain site, is surrounded by mountains, and thus lacks anthropogenic local emission sources. For this reason, the ground-level PM_10_ concentration is typically higher at the urban (Seoul) centre (58.2 ± 33.0 μg m^−3^) than at Mt. Gwan-ak (50.6 ± 41.0 μg m^−3^) by approximately 7–8 μg m^−3^ (Fig. 2(b)). Here the values in parentheses are the mean ± one standard deviation estimated during the study period (March-May in 2016). Despite the lack of local emissions, residential, commercial, and industrial pollutants emitted in Seoul, as well as those advected from China and other regions, yield higher PM_10_ concentrations at Mt. Gwan-ak. In Fig. 2(b,c), the measured PM_10_ concentration at Mt. Gwan-ak is sometimes higher than the ground-level concentration at the urban centre by more than 50.0 μg m^−3^ mostly between 21:00 and 06:00 Local Sidereal Time (LST). During the high episode period on 23 April, 2016, daily average of PM_10_ levels at Mt. Gwan-ak were recorded to be 262.0 μg m^−3^, showing a significant contrast against 181.0 μg m^−3^ recorded for the urban centre. The days on which the difference in the concentration exceeding 50 μg m^−3^ between the urban centre and Mt. Gwan-ak was noted, and were selected to determine the cause behind the high nocturnal concentrations at the mountain site.

The selected days were 15–16 March, 6 April, 8 April, 22–23 April, 4 May, 7 May, and 26 May in 2016. Asian dust was observed on 22–23 April and 7 May, whereas haze was observed on all the other days according to the weather records for Seoul (refer to http://www.weather.go.kr/weather/climate/past_cal.jsp). In an effort to identify the cause of the vertical PM_10_ concentration gradient, the backscattering coefficient signal of aerosol lidar, which identifies the vertical structure of atmospheric particles, was employed for the selected days. Subsequently, the atmospheric vertical structures and corresponding pollutant distributions were examined accordingly. In Seoul metropolitan area during the study period, a total of 7 days for which the warning was reported were the following: March/6, April/8–10, April/23, and May/7; March/6 was not selected in the current study because of the missing lidar measurements.

### Vertical structures of PM_10_ over Seoul Metropolitan Area

**Figure 3:**
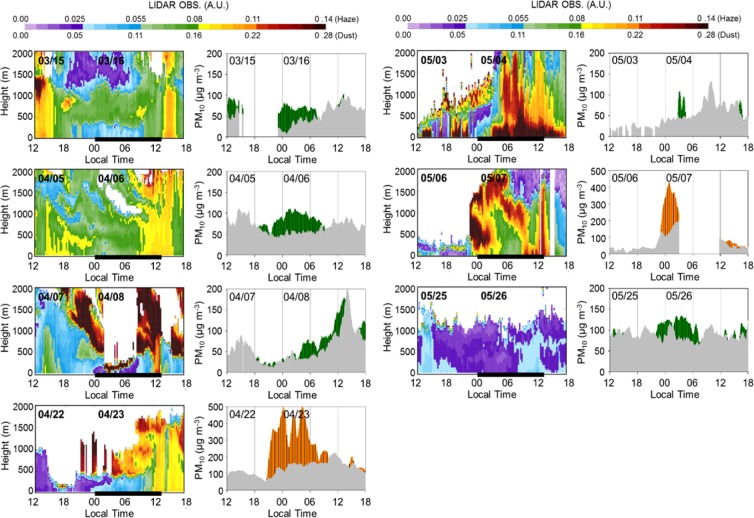
(Left) Measured aerosol backscattering coefficient distribution at Seoul National University (near Mt. Gwan-ak) on each selected day, (right) PM_10_ concentrations measured over time at Mt. Gwan-ak and near the ground, wherein the time period is the same as that in the aerosol backscattering coefficient graph on the left. The green and orange bars indicate the PM_10_ concentration levels at Mt. Gwan-ak under the influence of haze and Asian dust, respectively, whereas the grey bars denote the PM_10_ concentration levels at the ground surface in Seoul. Two different colour scales at the top, upper for haze and below for Asian Dust, are presented.

The comparison of PM_10_ concentration distributions in Seoul city centre and Mt. Gwan-ak as well as aerosol lidar backscattering coefficient distributions measured at Seoul National University, located near Mt. Gwan-ak, shows higher concentration levels at Mt. Gwan-ak from 18:00 to 06:00 LST of next day. The aerosol lidar measurements also display a high concentration plume at 500 m and above (Fig. 3). In particular, a layer with high backscattering coefficient was observed at 500 m and above in the presence of Asian dust between 21:00 LST of 22 April and 09:00 LST of 23 April. The vertical mixing of pollutants emitted at low altitudes was thereafter invigorated by the development of the ABL after sunrise as the ground surface heated up, causing the backscattering coefficient to increase from low to high altitudes. At noon, the sufficiently developed ABL mixed with the PM_10_ plume located approximately 1 km above the ground, resulting in a high PM_10_ concentration level from high to low altitudes according to both PM_10_ concentration measurements and aerosol lidar data. This phenomenon became more pronounced in the presence of Asian dust on 6 and 7 May, when the Asian dust plume observed at 1–2 km above the ground gradually intruded into the upper layer at approximately 1 km above the ground toward the ground surface, starting at 09:00 LST on 7 May.

In addition to Asian dust, the upper aerosol layer was observed from the night of 15 March until the morning of 16 March. It began to mix with the lower layer, starting at approximately noontime. Although the difference between the upper and lower layers was not apparent on the night of 5 April, the backscattering coefficient was relatively low at night near the ground, whereas aerosols were found to be distributed across several layers at 500 m and above (Fig. 3). Furthermore, the aerosol distribution signals were observed at 500 m and above both on the night of 7 April and at dawn on 8 April, and relatively low values were observed near the ground at these times. A layer with a relatively high aerosol distribution signal was also identified as an isolated layer at 500 m above ground level on the night of 25 May, although the PM_10_ concentration was not very high, as indicated in Fig. 3.

The vertical aerosol concentration difference decreased when aerosols that were present as isolated layers during nighttime at 500 m and above began to mix with air masses in the lower layer as the vertical mixture induced by the development of the lower atmospheric boundary from 09:00 LST met the upper aerosol layer.

**Figure 4:**
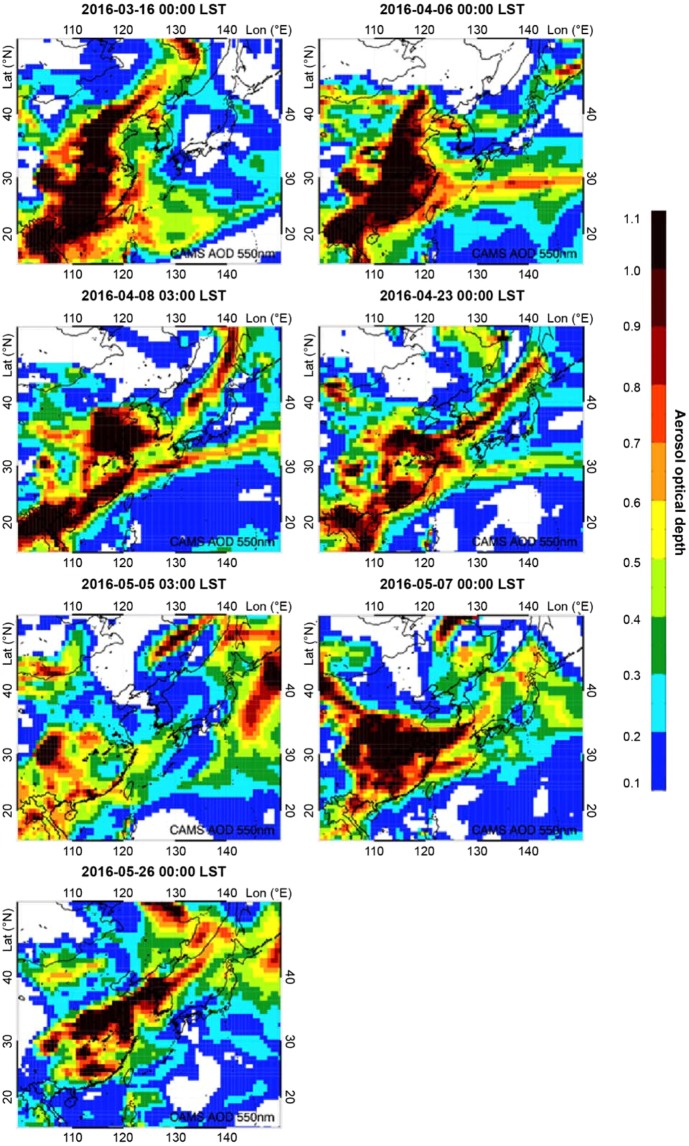
550-nm aerosol optical depth (AOD) spatial distributions of CAMS aerosol reanalysis data when high pollutant concentrations were observed at Mt. Gwan-ak for the selected days.

In the case of aerosol lidar measurements, however, identification of the horizontal movement of high concentration aerosol layer is rather challenging as the data yield the vertical aerosol distribution over time at fixed measurement locations. Notably, highly concentrated aerosol distributions initially arose from the surface or from lower altitudes, and then gradually mixed in higher altitudes as a result of the growing ABL. It is thus difficult to distinguish, solely based on aerosol lidar and PM_10_ concentrations measured at two locations, whether the phenomenon noted above is driven by such highly concentrated aerosol distributions prevailing above the nocturnal boundary layer (NBL) as its altitude decreases overnight, or by the long-range pollutants intruding into Seoul via streams at high altitudes. The horizontal aerosol distribution derived from the chemical transport model assimilated with observations of chemical compositions was evaluated to identify the path of the high concentration PM_10_ plume. The spatial distribution of total AOD in the 550 nm wavelength range of the Copernicus Atmosphere Monitoring Service (CAMS) reanalysis data provided by European Centre for Medium-Range Weather Forecasts (ECMWF) is shown in Fig. 4. According to the figure, high AOD values (>0.5) were observed from China all the way to the west of the Korean Peninsula over the Yellow Sea when high nocturnal concentrations were measured at Mt. Gwan-ak on each selected day. It is also noteworthy that in some cases in this study, two or three instances of extremely high AOD (>1.1) were observed as being present between the Yellow Sea and the Seoul Metropolitan Area on 23 April, 7 May, and 26 May. The high nocturnal aerosol concentrations over Seoul were thus attributed to the long-range pollutant intrusion affected by aerosol plumes generated in China.

### Influence of the nocturnal boundary layer/convective boundary layer

**Figure 5:**
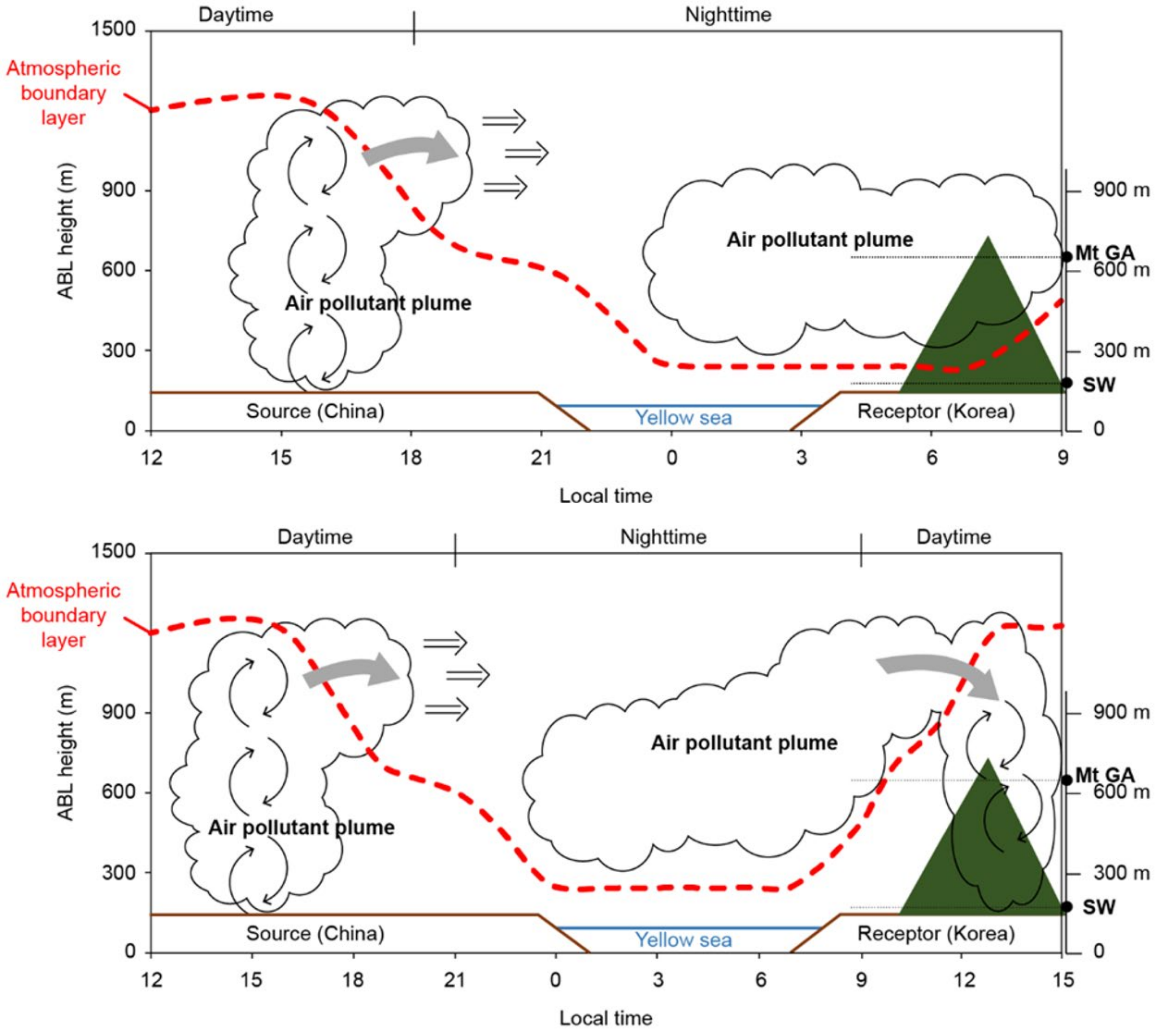
Illustration of long-range transboundary pollutant intrusions from China into the Korean peninsula under ABL variations in the emission source region (China), over the Yellow Sea, and in the receptor region (the Korean peninsula). “Mt. GA” denotes Mt. Gwan-ak. The transported air pollutant plume stays above nocturnal boundary layer at the receptor region without influencing the surface concentrations (top). As ABL grows after sunrise, the transported pollutants intrude into the receptor’s ABL by entrainment and vertical mixing (bottom).

The horizontal and vertical behaviours of aerosols measured and predicted for Seoul, Korea, verify the intrusion of pollutants generated in China via the upper layer at an altitude of 500 m and above instead of near the ground surface. The pollutants remain as a decoupled layer above the ABL, especially when they remain overnight, until the lower vertical mixing gradually occurs after sunrise and the mixing layer grows to transport the pollutants. High pollutants in the upstream area (i.e., emitted from cities and industrial plants) travel along the upper layer because they intrude through the varying land-sea-land surface conditions as the Yellow Sea is located between China and the Korean peninsula. These conditions are responsible for the long-range transport of the pollutants. Moreover, the vertical mixing induced in the emission region in the upstream area carries the local pollutants to higher altitudes. They then flow toward the Yellow Sea with the westerly and north-westerly winds. Most pollutants within the MBL are deposited at this time while the incoming pollutants above the MBL are diverted to leeward regions. Subsequently, the pollutants are believed to intrude into the Korean peninsula at high altitudes rather than near the ground surface as they are transported over the westerly MBL. When the pollutants that flow along the upper layer over the Yellow Sea reach Korea at nighttime, they remain above the lower NBL and do not mix with the air mass near the ground. The concentration levels at the city centre in Seoul are lower than those at Mt. Gwan-ak, whereas Seoul shows gradually higher levels when the long-range transported pollutants from China, which intrude into Seoul in the daytime gradually reach the lower layer after mixing at the top of the sufficiently developed ABL. The horizontal/vertical behaviour as well as the long-range transport and intrusion of pollutants generated in China are illustrated in Fig. 5.

During winter and spring, the air quality in the leeward regions in the Korean peninsula often degrades due to pollutants emitted in China, leading to severe public health concerns. As a result, the provision of prognostic air quality information to the public, by accurate identification of the intrusion period and mechanism of long-range transported pollutants, is imperative. This study suggests that long-range transported pollutants may have a greater impact on surface air quality in daytime than nighttime, and it aims to facilitate more accurate air quality forecasts for the leeward areas. In particular, many studies have demonstrated that when the altitude of the ABL is low, vertical mixing does not occur, and thus, the observed surface concentrations are high mainly due to the weaker dilution process^24–28^. In this study, we highlighted the inverse correlation between the boundary layer mixing process and ground-level particulate matter concentrations, suggesting that the development of the ABL can play a positive role by increasing the surface concentration by introducing inflowing pollutants from the upper atmosphere when the ABL height increases.

**Figure 6:**
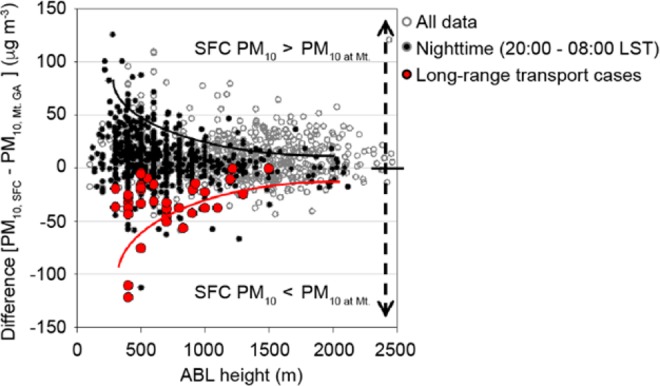
PM_10_ concentration difference between the ground surface and at Mt. Gwan-ak versus ABL height in Seoul between March and May of 2016. The grey open, black closed, and red closed circles indicate all the data (over the entire period), the measured/estimated values during nighttime (20:00–08:00 LST), and the measured/estimated values for the long-range transport cases selected in this study, respectively.

Figure 6 shows the correlation between the estimated ABL height and the concentration differences between the upper and lower altitudes. Here, ABL heights were estimated by observing the vertical profiles of attenuated backscatter^29–31^; these showed a seasonal average of 1182 (±540) m and 548 (±180) m during day and night, respectively, during spring 2016. As denoted in Fig. 6, the low NBL with the higher surface concentrations, interferes with the mixing of pollutant concentrations above and at the surface level, thus maintaining higher concentrations at the surface and lower ones at higher altitudes (PM_10_ at surface > PM_10_ at Mountain). This implies little influence of intrusion from above when meteorological conditions are stagnant. However, in the case of long-range transport, a significant difference in concentrations between the upper and surface levels with higher upper and lower surface PM_10_ concentration patterns was confirmed, indicating that the upper layer pollutants do not affect the lower layer at night. In both cases, it was confirmed that the concentration difference between the upper and lower layers decreased as the altitude of the ABL increased during daytime.

## Discussion

PM_10_ warnings/alerts were issued in Seoul six times in total in 2016, and the following four such cases (67% of the total) are discussed here. They are characterised with high pollutant concentrations at Gwan-ak Mountain, which gradually affected the ground level concentrations. Thus, more than 67% of the deterioration in air quality over the Korean Peninsula was attributed to the inflow of the long-range transboundary of moving pollutant plumes. Detailed analyses of long-term 3-dimensional simulations/observations of other cases would be needed for more robust information on such intrusions, including the arrival heights and intrusion times under various meteorological conditions. This information can be used for the identification and prediction of starting and ending times of highly concentrated plumes.

In addition, understanding the vertical behaviours of these pollutant plumes can assist future studies on chemical reactions occurring in the upper and lower atmospheric layers^32,33^ of the Korean peninsula. Intensive high-resolution vertical measurements of atmospheric chemical compositions during nighttime as well as daytime are required to analyse the physicochemical behaviours of the pollutants corresponding to daylight/nocturnal chemical reactions.

## Methods

### Observation data

A ground-based surface PM_10_ monitoring system operated by the Korea Meteorological Administration (KMA) provided PM_10_ concentrations measured by an aerodynamic particle sizer (APS) at 5-min intervals over dozens of stations nationwide. In this study, the comparisons with PM_10_ concentrations at different sites, namely Mt. Gwan-ak (37.44525°N, 126.96401°E, 622.38 m) and a ground-level site located within the urban centre (Song-wol, 37.57141°N, 126.96579°E, 85.8 m; Fig. 1), were conducted to estimate the vertical inflow structures and intrusions of aerosol plumes over the leeward areas.

In addition to PM_10_ concentration measurements, real-time aerosol lidar observations at Seoul National University (37.460°N, 126.950°E, 300 m, near Mt. Gwan-ak) were used to analyse detailed vertical profiles of aerosol and atmospheric vertical structures related to evolution of the ABL. The aerosol lidar observations provided information on the vertical behaviours of the aerosols using the vertical profiles of range-correlated backscatter signals and depolarization ratios measured with 532 nm and 1064 nm wavelength lasers. However, the aerosol lidar observations were lacking in terms of missing values for extremely high aerosol pollution incidents. Detailed information on the aerosol lidar instrument appears at http://www.kalion.kr/intro/kalionRidar.

We also used ABL height data estimated from the vertical profiles of attenuated backscatter obtained by a ceilometer (Model CL51, Vaisala, Finland) at an urban meteorological observation system station located in the eastern part of the city of Seoul (37.59°N, 127.08°E, 45 m)^29–31^. The ceilometer provided the vertical distributions of two-way attenuated backscatter by means of a 910 nm wavelength laser as well as three levels of cloud base height. The attenuated backscatter with a vertical resolution of 10 m up to 15 km, and a temporal resolution of 1 min depended on the aerosol concentration sensitive to the 910 nm wavelength and the vertical aerosol profile represented the boundary layer structure. Thus, the boundary layer features, such as mixing layer and residual layer, could be visually determined from the time–height cross sections of the attenuated backscatter.

### Model data

The Copernicus Atmosphere Monitoring Service (CAMS) reanalysis is a global reanalysis data set of atmospheric composition produced by the European Centre for Medium-Range Weather Forecasts (ECMWF). It consists of 3-dimensional time-consistent atmospheric composition fields^34^. Several satellite retrievals such as total CO columns, tropospheric NO_2_ columns, AOD, and total and partial columns of ozone were assimilated for CAMS reanalysis with ECMWF’s Integrated Forecasting System. This dataset has a horizontal resolution of approximately 80 km and is available for 2003–2016 at https://apps.ecmwf.int/data-catalogues/cams-reanalysis/.

### Graphics software

All contour plots were produced using Interactive Data Language (IDL, https://www.harrisgeospatial.com/Software-Technology/IDL). All bar charts and scatter plots were plotted using the Sigma Plot program (http://www.sigmaplot.co.uk/products/sigmaplot/sigmaplot-details.php).

## Data Availability

The CAMS reanalysis data generated during and/or analysed during the current study are available in the ECMWF website- https://apps.ecmwf.int/data-catalogues/cams-reanalysis/. All measured data related to this paper are available from the corresponding author on reasonable request.
